# Mechanisms Underlying Insulin Deficiency-Induced Acceleration of β-Amyloidosis in a Mouse Model of Alzheimer's Disease

**DOI:** 10.1371/journal.pone.0032792

**Published:** 2012-03-05

**Authors:** Latha Devi, Melissa J. Alldred, Stephen D. Ginsberg, Masuo Ohno

**Affiliations:** 1 Center for Dementia Research, Nathan Kline Institute, Orangeburg, New York, United States of America; 2 Department of Psychiatry, New York University Langone Medical Center, New York, New York, United States of America; 3 Department of Physiology and Neuroscience, New York University Langone Medical Center, New York, New York, United States of America; Massachusetts General Hospital/Harvard Medical School, United States of America

## Abstract

Although evidence is accumulating that diabetes mellitus is an important risk factor for sporadic Alzheimer's disease (AD), the mechanisms by which defects in insulin signaling may lead to the acceleration of AD progression remain unclear. In this study, we applied streptozotocin (STZ) to induce experimental diabetes in AD transgenic mice (5XFAD model) and investigated how insulin deficiency affects the β-amyloidogenic processing of amyloid precursor protein (APP). Two and half months after 5XFAD mice were treated with STZ (90 mg/kg, i.p., once daily for two consecutive days), they showed significant reductions in brain insulin levels without changes in insulin receptor expression. Concentrations of cerebral amyloid-β peptides (Aβ40 and Aβ42) were significantly increased in STZ-treated 5XFAD mice as compared with vehicle-treated 5XFAD controls. Importantly, STZ-induced insulin deficiency upregulated levels of both β-site APP cleaving enzyme 1 (BACE1) and full-length APP in 5XFAD mouse brains, which was accompanied by dramatic elevations in the β-cleaved C-terminal fragment (C99). Interestingly, BACE1 mRNA levels were not affected, whereas phosphorylation of the translation initiation factor eIF2α, a mechanism proposed to mediate the post-transcriptional upregulation of BACE1, was significantly elevated in STZ-treated 5XFAD mice. Meanwhile, levels of GGA3, an adapter protein responsible for sorting BACE1 to lysosomal degradation, are indistinguishable between STZ- and vehicle-treated 5XFAD mice. Moreover, STZ treatments did not affect levels of Aβ-degrading enzymes such as neprilysin and insulin-degrading enzyme (IDE) in 5XFAD brains. Taken together, our findings provide a mechanistic foundation for a link between diabetes and AD by demonstrating that insulin deficiency may change APP processing to favor β-amyloidogenesis via the translational upregulation of BACE1 in combination with elevations in its substrate, APP.

## Introduction

Alzheimer's disease (AD) is a devastating neurodegenerative disorder and the most common form of dementia among the elderly population. Although the cause of AD in sporadic forms has not been completely determined, there is increasing consensus that accumulation of the amyloid-β (Aβ) peptide plays a central role in triggering a cascade ultimately leading to profound neuronal death and memory defects [1], [2]. Importantly, a number of risk factors have been identified that may shed light on the molecular mechanisms underlying the development of sporadic AD; in particular, recent evidence suggests a close relationship between sporadic AD and diabetes mellitus [3]–[5]. Consistent with the observation that patients with type 2 diabetes characterized by insulin resistance are at an increased risk of getting AD, not only reductions in insulin levels and insulin receptor expression but also deficient downstream signaling pathways have been reported to occur in brains of AD patients [6]–[9]. Furthermore, clinical data are available on the association between AD and type 1 diabetes resulting from hypoinsulinemia [10]. Therefore, it seems reasonable to hypothesize that sporadic AD represents a form of diabetes that selectively involves the brain and has the disturbed insulin signaling pathway in common with type 1 and type 2 diabetes mellitus [11]. This hypothesis is supported by recent findings that analogue compounds for the incretin hormone glucagon-like peptide-1 (GLP-1), which facilitate endogenous insulin release and are used to treat type 2 diabetes, reduce Aβ accumulation and rescue impairments in hippocampal synaptic plasticity and spatial learning and memory in transgenic mouse models of AD [12]–[14]. In a clinical setting, treatments with insulin in combination with other antidiabetic medication are shown to lower plaque load and benefit cognitive function in AD patients with diabetes [15], [16]. Moreover, it has been demonstrated that soluble Aβ oligomers produce a loss of neuronal surface insulin receptors and directly interfere with the insulin signaling pathway [17]–[20].

To further investigate an association between neuronal insulin dysfunction and Aβ accumulation in AD, streptozotocin (STZ), an agent that selectively destroys insulin-secreting pancreatic β cells and thereby causes insulin depletion, has been applied to AD transgenic mice overexpressing mutant human amyloid precursor protein (APP) [5]. Interestingly, these studies have revealed that STZ-induced cerebral insulin deficiency in APP transgenic mice exacerbates the development of AD-like phenotypes such as β-amyloidosis and memory impairments [21]–[23]. Taken collectively, different lines of experimental data suggest a link between diabetes and AD in the pathogenesis of disease; however, the underlying mechanism is poorly understood.

In this study, we investigated the mechanisms by which insulin deficiency may accelerate AD progression in the 5XFAD transgenic mouse model. 5XFAD mice co-overexpress human APP and presenilin 1 (PS1) harboring five familial AD (FAD) mutations [24]–[26]. 5XFAD mice begin to develop visible amyloid deposition as early as 2 months of age and exhibit memory declines on hippocampus-dependent behavioral tasks between 4–6 months concomitant with moderate Aβ accumulation and impaired synaptic physiology at Schaffer collateral-CA1 pathways [24], [25], [27]–[29]. For our study, STZ was administered to young 5XFAD mice (1.5 months old) that have not yet developed amyloid pathology, and we compared levels of key molecules involved in β-amyloidosis between STZ- and vehicle-treated subjects at 4 months of age. The molecules tested include β-site APP cleaving enzyme 1 (BACE1), a disintegrin and metalloproteinase 10 (ADAM10) and PS1 responsible for the processing of APP, full-length APP, its β- and α-cleavage products, and Aβ-degrading enzymes such as neprilysin and insulin-degrading enzyme (IDE). We demonstrate that insulin deficiency facilitates cerebral β-amyloidogenesis in 5XFAD mouse brains accompanied by significant elevations in BACE1 and APP expression in the absence of changes in levels of α-, γ-secretase or Aβ-degrading enzymes. The results also suggest that translational mechanisms through phosphorylation of eukaryotic initiation factor-2α (eIF2α) may underlie the upregulation of BACE1 associated with insulin-deficient diabetes.

## Results

### Insulin deficiency facilitates Aβ accumulation in 5XFAD mice

**Figure 1:**
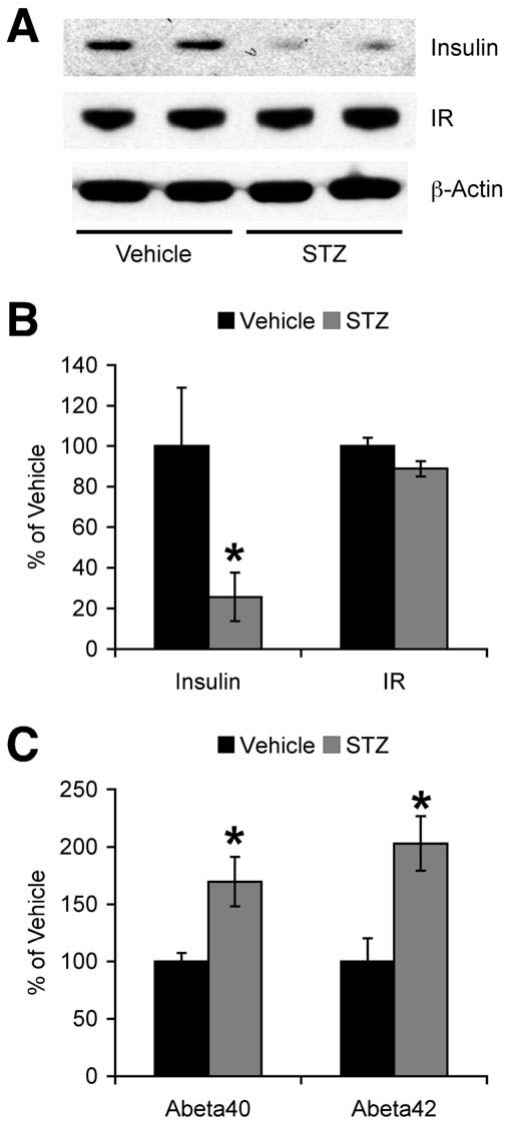
Effects of STZ-induced insulin deficiency on Aβ accumulation in 5XFAD mice. (*A*) Western blot analysis of hemibrain lysates from vehicle- and STZ-treated 5XFAD mice. (*B*) Immunoreactive bands for insulin and insulin receptor (IR) were quantified and expressed as the percentage of vehicle-treated 5XFAD levels (*n* = 4–7 mice per group). STZ treatments significantly reduced cerebral insulin levels without affecting IR in 5XFAD mice (*p<0.05 vs. vehicle). (*C*) Levels of total Aβ40 and Aβ42 were quantified by sandwich ELISAs of guanidine extracts of hemibrain samples and expressed as the percentage of vehicle-treated 5XFAD levels (*n* = 3–5 mice per group). Aβ40 and Aβ42 levels were significantly higher in brains of STZ-treated 5XFAD mice (*p<0.05 vs. vehicle). All data are presented as mean ± SEM.

To examine the effects of insulin deficiency on β-amyloidosis, 5XFAD mice at 1.5 months of age were administered with STZ (90 mg/kg, i.p.) once daily for two consecutive days after overnight fast. Two and half months later, hemibrain samples were collected from STZ- and saline vehicle-treated subjects. First, immunoblot analysis of brain homogenates demonstrated that insulin levels were significantly reduced in STZ-treated 5XFAD mice compared with saline-treated 5XFAD controls (*F*(1,9) = 7.98, *p*<0.05), while STZ treatments did not affect insulin receptor expression (Fig. 1A, B). Next, sandwich ELISAs were performed to compare Aβ levels in STZ- and vehicle-treated 5XFAD mice (Fig. 1C). Consistent with previous findings [21]–[23], STZ-induced insulin deficiency significantly increased cerebral levels of Aβ40 (*F*(1,7) = 7.49, *p*<0.05) and Aβ42 (*F*(1,6) = 8.56, *p*<0.05) in 5XFAD mice.

### Insulin deficiency elevates BACE1 and full-length APP in 5XFAD mice

**Figure 2:**
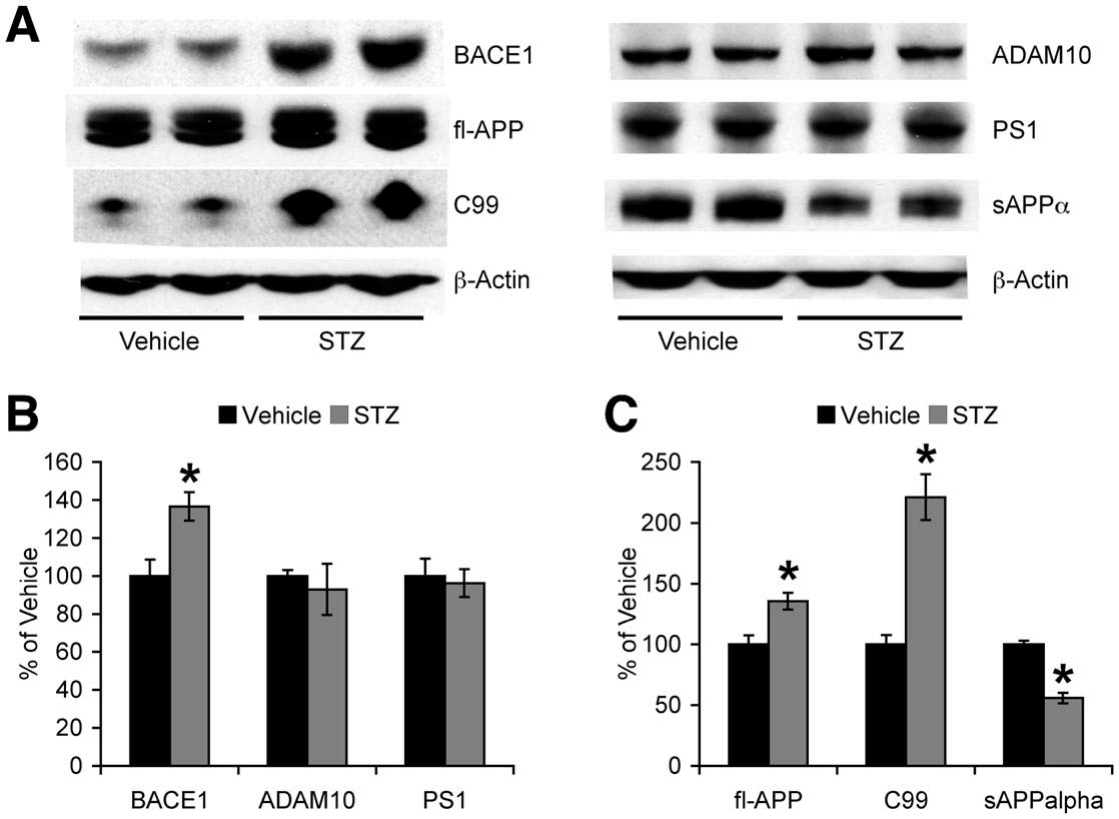
Effects of STZ-induced insulin deficiency on APP processing in 5XFAD mice. (*A*) Western blot analysis of hemibrain lysates from vehicle- and STZ-treated 5XFAD mice. Immunoreactive bands for secretases involved in the APP cleavage (*B*), full-length APP (fl-APP) and its metabolites (*C*) were quantified and expressed as the percentage of vehicle-treated 5XFAD levels (*n* = 4–7 mice per group). BACE1, fl-APP and C99 levels were significantly increased, while sAPPα levels were significantly reduced in STZ-treated 5XFAD mice (*p<0.05 vs. vehicle). All data are presented as mean ± SEM.

To address the mechanisms underlying the acceleration of Aβ accumulation in STZ-induced diabetic 5XFAD mouse brains, we first investigated changes in the β-amyloidogenic processing of APP (Fig. 2). Immunoblot analysis demonstrated that STZ-induced insulin deficiency elevated protein levels of the β-secretase BACE1 in 5XFAD mice (Fig. 2A). Quantitative analysis revealed that BACE1 expression in brains of STZ-treated 5XFAD mice was significantly higher than that of vehicle-treated 5XFAD mice (*F*(1,9) = 9.49, *p*<0.05) (Fig. 2B). It should be noted that baseline levels of BACE1 in vehicle-treated groups were indistinguishable between 5XFAD and wild-type control mice at 4 months of age (data not shown), although BACE1 expression increases with age (≥9 months) in 5XFAD mouse brains [30], [31], Meanwhile, STZ treatments did not significantly affect levels of ADAM10 and PS1 associated with α- and γ-secretase activities, respectively, in 5XFAD mice (Fig. 2B). Therefore, the change was specific to β-secretase in insulin-deficient 5XFAD mice.

In addition to increases in BACE1 expression, STZ treatments also significantly elevated levels of full-length APP, a substrate of BACE1, in 5XFAD mice (*F*(1,9) = 10.66, *p*<0.05) (Fig. 2C). Consistent with these changes, levels of the β-cleaved C-terminal fragment of APP (C99) were dramatically elevated (*F*(1,9) = 21.73, *p*<0.05), while levels of the secreted ectodomain of APP formed by α-secretase cleavage (sAPPα) were significantly reduced (*F*(1,9) = 51.26, *p*<0.05) in STZ-treated 5XFAD mouse brains (Fig. 2C). Together, these data indicate that STZ-induced insulin deficiency alters APP processing to promote the β-amyloidogenic pathway through the upregulation of BACE1 and APP expression.

### Mechanisms by which insulin deficiency elevates BACE1 in 5XFAD mice

**Figure 3:**
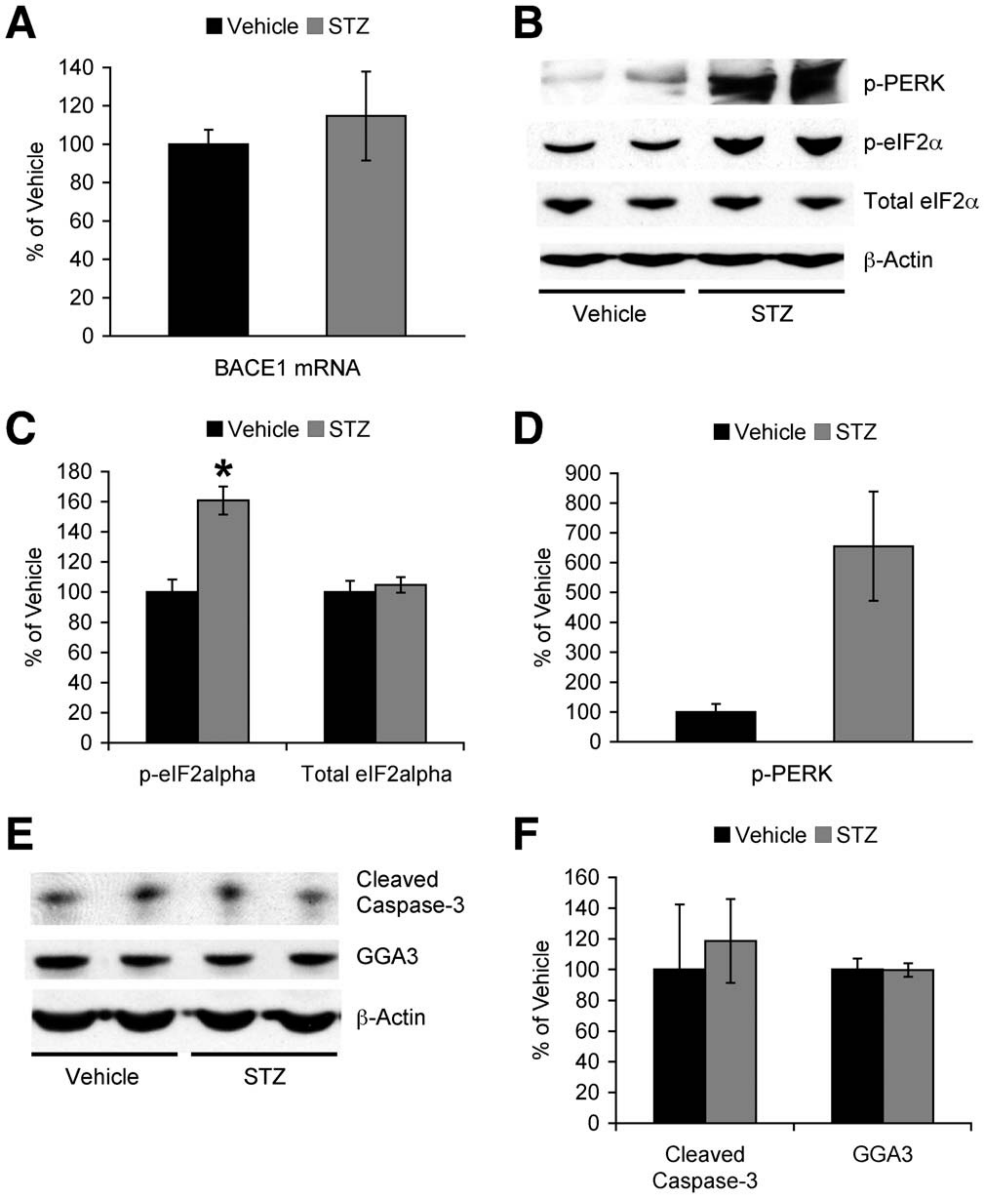
Mechanisms by which STZ-induced insulin deficiency elevates BACE1 levels in 5XFAD mice. (*A*) Real-time qPCR revealed no difference in BACE1 mRNA levels between STZ- and vehicle-treated 5XFAD mouse brains (*n* = 4–5 mice per group). (*B, E*) Western blot analysis of hemibrain lysates from vehicle- and STZ-treated 5XFAD mice. Immunoreactive bands for phosphorylated eIF2α (p-eIF2α) and total eIF2α (*C*), phosphorylated PERK (p-PERK) (*D*), and the 17-kDa fragment of activated caspase-3 and GGA3 (*F*) were quantified and expressed as the percentage of vehicle-treated 5XFAD levels (*n* = 4–7 mice per group). Levels of p-eIF2α, but not those of total eIF2α, were significantly elevated in STZ-treated 5XFAD mice (*p<0.05 vs. vehicle). STZ treatments also dramatically increased p-PERK levels in 5XFAD mice (*p* = 0.05), while cleaved caspase-3 and GGA3 levels were indistinguishable between STZ- and vehicle-treated subjects. All data are presented as mean ± SEM.

We next investigated whether transcriptional and/or post-transcriptional mechanisms may underlie the BACE1 elevation found in STZ-induced diabetic 5XFAD mice (Fig. 3). qPCR analysis of brain homogenates revealed that BACE1 mRNA levels were not significantly different between STZ- and vehicle-treated 5XFAD mice (Fig. 3A). Recent studies including ours demonstrate that phosphorylation of the translation initiation factor eIF2α (phospho-eIF2α) plays an important role in mediating the post-transcriptional upregulation of BACE1 in sporadic AD and 5XFAD mice at advanced stages of disease with massive amyloid pathology [31]–[33]. Therefore, we tested the possibility that the phospho-eIF2α pathway may be involved in the upregulation of BACE1 in STZ-treated 5XFAD mice (Fig. 3B). In parallel with elevations in BACE1 expression, STZ-induced insulin deficiency resulted in significant increases in phospho-eIF2α levels (*F*(1,9) = 18.78, *p*<0.05) without affecting total eIF2α levels in 5XFAD mouse brains (Fig. 3C). Moreover, we found that STZ treatments activated PKR-endoplasmic reticulum-related kinase (PERK: an eIF2α kinase) in 5XFAD mouse brains, as measured by a dramatic increase in a phosphorylated form of PERK (*F*(1,9) = 4.97, *p* = 0.05) (Fig. 3D). Therefore, the results suggest that translational mechanisms through activation of the eIF2α phosphorylation pathway rather than transcriptional mechanisms may account for the elevation of BACE1 expression in brains of STZ-induced diabetic 5XFAD mice.

We further examined whether changes in the BACE1-degrading pathway may be involved in the BACE1 elevation in STZ-treated 5XFAD mouse brains (Fig. 3E). Recent evidence indicates that BACE1 protein stability can be modulated by the lysosomal degradation pathway; in particular, caspase-3-dependent cleavage of Golgi-localized γ-ear-containing ARF-binding protein 3 (GGA3) is proposed to reduce BACE1 trafficking to the lysosome and thereby cause BACE1 elevation in AD [34]–[37]. In this study, STZ treatments did not induce caspase-3 activation as assessed by increases in its 17-kDa fragments or reductions in GGA3 levels in 5XFAD mice (Fig. 3F). Therefore, it is unlikely that alteration of the GGA3-mediated BACE1 degradation mechanism may underlie the upregulation of BACE1 associated with insulin deficiency in 5XFAD mice.

### Insulin deficiency does not affect neprilysin or IDE levels in 5XFAD mice

**Figure 4:**
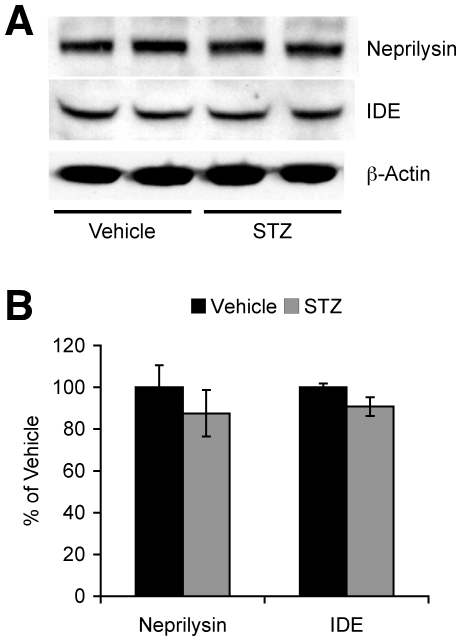
Effects of STZ-induced insulin deficiency on neprilysin and IDE levels in 5XFAD mice. (*A*) Western blot analysis of hemibrain lysates from vehicle- and STZ-treated 5XFAD mice. (*B*) Immunoreactive bands for neprilysin and IDE were quantified and expressed as the percentage of vehicle-treated 5XFAD levels (*n* = 4–7 mice per group). There was no difference in neprilysin or IDE levels between STZ- and vehicle-treated 5XFAD mice. All data are presented as mean ± SEM.

In addition to the facilitation of β-amyloidogenesis through BACE1 and APP elevations, it is possible that changes in the Aβ metabolism may also contribute to increases in Aβ40 and Aβ42 levels in STZ-treated 5XFAD mice. Therefore, we examined whether STZ treatments may affect neprilysin and/or IDE, key enzymes responsible for the degradation and clearance of Aβ peptides [38], [39], in 5XAFD mice (Fig. 4A). However, levels of neprilysin and IDE were indistinguishable between STZ- and vehicle-treated 5XFAD mouse brains (Fig. 4B), suggesting that increased levels of Aβ accumulation in insulin-deficient diabetic 5XFAD mice do not result from alterations in these Aβ-degrading enzymes.

## Discussion

Consistent with epidemiological investigations showing that diabetes is an important risk factor for sporadic AD, a growing body of evidence indicates defective insulin signaling in AD brains [6]–[9]. In this study, insulin deficiency evoked by STZ administration in the pre-pathological stage of 5XFAD mice (1.5 months of age) accelerated the subsequent Aβ accumulation in brain, suggesting that insulin dysfunction may not only be a consequence of AD pathology but also play a causal role in triggering the disease process. In other transgenic mouse models of AD, STZ-induced cerebral insulin depletion has also been reported to aggravate AD-like traits such as amyloid plaques, tau phosphorylation, neurofibrillary tangles, and spatial memory deficits in the Morris water maze or Barnes maze task [21]–[23], [40]. We confirmed and extended the previous findings by determining the mechanisms by which STZ-induced insulin-deficient diabetes aggravates β-amyloidosis in 5XFAD transgenic mice.

We first demonstrated that protein levels of BACE1, a key enzyme that initiates the production of Aβ peptides from their parent molecule APP, are significantly elevated in STZ-treated 5XFAD mice. In contrast, STZ treatments did not change levels of ADAM10 and PS1 involved in the α- and γ-secretase cleavage of APP, respectively, in 5XFAD mice. Therefore, BACE1 upregulation represents a crucial mechanism linking insulin deficiency to enhanced Aβ40 and Aβ42 accumulation in brain. However, BACE1 mRNA levels were not significantly affected by STZ treatments in 5XFAD mice, suggesting that transcriptional mechanisms may not account for the BACE1 elevation associated with insulin-deficient diabetes. It has been reported that increases in phosphorylation of the translation initiation factor eIF2α may underlie the post-transcriptional upregulation of BACE1 in brains of sporadic AD and advanced pathological phases of 5XFAD transgenic mice (≥9 months of age) [31], [33]. In the present study, baseline levels of BACE1 in vehicle-treated 4-month-old 5XFAD mice, which show modest Aβ deposition [24], were indistinguishable from wild-type control levels, while STZ treatments triggered BACE1 elevations concomitant with increased levels of phosphorylated eIF2α in 5XFAD mouse brains. Therefore, the results indicate that eIF2α phosphorylation-dependent translational upregulation of BACE1 in response to insulin deficiency may represent an important molecular mechanism by which diabetes accelerates β-amyloidogenesis before significant Aβ deposition occurs during the incipient stage of sporadic AD. This idea is supported by our recent observation that the increase in phospho-eIF2α evoked by Sal 003, a specific inhibitor of its phosphatase, elevates BACE1 protein levels in younger 5XFAD mice that have not yet showed BACE1 upregulation under normal conditions [31].

What mechanisms may underlie a link between insulin signaling and the phosphorylation of eIF2α? It is reported that an increase in phosphorylated eIF2α occurs in hippocampal neurons of rats exposed to transient cerebral ischemia, while insulin administration can almost completely eliminate the phosphorylation of eIF2α in this model [41]. Therefore, it is conceivable that insulin may downregulate eIF2α kinase and/or upregulate eIF2α phosphatase. In this study, we found that insulin deficiency produces robust activation of PERK, a major eIF2α kinase, in line with elevated levels of phospho-eIF2α in STZ-treated 5XFAD mice. This is consistent with the observation showing that mRNA and protein levels of PERK are significantly upregulated in STZ-injected rats [42]. Moreover, expression of dominant-negative PERK is shown to block energy deprivation-induced increases of both eIF2α phosphorylation and BACE1 in HEK293 cell line cultures [33]. Although further study is required for the demonstration of a causal link, the present data suggest that the PERK-eIF2α phosphorylation pathway may, at least in part, play a role in mediating the BACE1 elevation associated with deficient insulin signaling in diabetic 5XFAD mice.

Are transcriptional or translational mechanisms responsible for increases in protein and activity levels of BACE1 in sporadic AD brains [43]–[45]? Some studies with postmortem human brains report elevations in BACE1 mRNA levels associated with sporadic AD [46], [47], while others show no changes in mRNA despite the increased levels of BACE1 activity and protein [48]–[51]. Interestingly, our previous study demonstrated that behavioral stress elevates BACE1 protein levels concomitant with increases in both BACE1 mRNA and a phosphorylated form of eIF2α in 5XFAD mice [52], while BACE1 expression is upregulated without increases in mRNA levels in the diabetic 5XFAD model in this study. Although further investigation is needed, it is interesting to argue that differences in environmental factors, which predominantly contribute to disease progression, may determine whether the transcriptional and/or translational mechanisms underlie BACE1 elevations in sporadic AD.

With regard to BACE1 stability, GGA3 has been proposed to play a crucial role in the transport of ubiquitinated BACE1 to the lysosomal degradation [34], [36]. GGA3 is a substrate for caspase-3 cleavage; therefore, under apoptotic conditions, GGA3 inactivation by caspase-3 leads to increased BACE1 protein stability [34], [35]. Importantly, GGA3 levels are significantly decreased in AD brains and inversely correlate with increased levels of BACE1 expression [34], [37]. However, the absence of caspase-3 activation and GGA3 reduction in our STZ-treated 5XFAD model indicates that the mechanism of BACE1 elevation associated with diabetic insulin deficiency is distinct from mechanisms mediated by depletion of the BACE1-sorting protein GGA3 during apoptosis.

In addition to BACE1 elevations, levels of the substrate APP were also upregulated in STZ-treated 5XFAD mice. Our result is consistent with a recent report showing that expression levels of full-length APP are increased by STZ treatments in the absence of changes in APP mRNA levels in APP/PS1 transgenic mice [23]. Therefore, it seems likely that post-transcriptional upregulation of both BACE1 and its substrate APP may work cooperatively, leading to significant acceleration of β-amyloidogenesis in brains of insulin-deficient diabetic AD mice.

In contrast, STZ treatments did not affect levels of the Aβ-degrading enzymes such as neprilysin and IDE in 5XFAD mouse brains. Our present results as well as others [23] indicate that the facilitation of Aβ production, which is accompanied by increases in the intermittent β-cleaved C-terminal fragment C99 resulting from elevated BACE1 and APP expression, may account for the exacerbation of Aβ accumulation in brains of STZ-induced diabetic AD transgenic mice. These findings are in contrast with a report showing that STZ-induced insulin deficiency caused reductions in IDE without affecting C99 levels in APP mice, suggesting that diabetes-associated exaggeration of β-amyloidosis may be a result of reduced Aβ degradation rather than increased Aβ generation [21]. This discrepancy may arise from differences in transgenic AD mouse models used for the experiments and the age tested or the extent to which STZ treatments reduce cerebral insulin levels. Further study will be required to address the relative contribution of increased generation and/or reduced degradation of Aβ to the enhanced β-amyloidosis in diabetic AD mice, with special focus on the relationship to alterations in insulin signaling pathways.

In conclusion, the results presented here demonstrate that STZ-induced insulin-deficient diabetes exacerbates Aβ accumulation by elevating expression levels of the β-secretase enzyme BACE1 and its substrate APP in the 5XFAD mouse model of AD. BACE1 elevations in diabetic 5XFAD mouse brains seem to be associated with translational upregulation through the PERK-eIF2α phosphorylation pathway rather than transcriptional mechanisms or changes in the GGA3-dependent lysosomal degradation. Our data support the hypothesis that deficient insulin signaling may represent a critical contributing factor in the acceleration of β-amyloidogenesis during the progression of sporadic AD and thus may be an important therapeutic target in AD treatments.

## Materials and Methods

### Animals

We used 5XFAD transgenic mice (Tg6799 line) that co-overexpress FAD mutant forms of human APP (the Swedish mutation: K670N, M671L; the Florida mutation: I716V; the London mutation: V717I) and PS1 (M146L; L286V) transgenes under transcriptional control of the neuron-specific mouse Thy-1 promoter [24]–[26]. 5XFAD lines (B6/SJL genetic background) were maintained by crossing hemizygous transgenic mice with B6/SJL F1 breeders (Taconic, Hudson, NY, USA). 5XFAD transgenic mice used were hemizygotes with respect to the transgene and non-transgenic wild-type littermate mice served as controls. Genotyping was performed by PCR analysis of tail DNA, as described [24]. Procedures were conducted in accordance with the National Institutes of Health Guide for the Care and Use of Laboratory Animals and approved by the Nathan Kline Institute Animal Care and Use Committee (Assignment number: AP2008-268).

### Induction of insulin-deficient diabetes

5XFAD and wild-type control mice at 1.5 months of age were fasted overnight and received an intraperitoneal injection of streptozotocin (STZ, Sigma-Aldrich, St Louis, MO, USA) at 90 mg/kg or saline vehicle once daily for two consecutive days. The mice were sacrificed at 4 months of age and brain samples were collected for analysis.

### Immunoblot analysis

Hemibrain samples were taken from the mice under deep isoflurane anesthesia and were snap-frozen for biochemical assays. For western blot analysis, each sample was homogenized in 5 volumes of modified RIPA buffer containing 150 mM NaCl, 50 mM Tris HCl (pH 8.0), 1 mM EDTA, 1% IGEPAL, 0.5% sodium deoxycholate, 0.1% SDS and protease/phosphatase inhibitor cocktail (Calbiochem, La Jolla, CA, USA), and centrifuged at 10,000 g for 10 min to remove any insoluble material. Protein concentrations were determined by a BCA protein assay kit (Pierce, Rockford, IL, USA), and 20–50 µg of protein was run on 4–12% NuPAGE gels (Invitrogen, Carlsbad, CA, USA) and transferred to nitrocellulose membranes. After blocking, membranes were probed with anti-insulin (1∶500, sc-9168, Santa Cruz Biotechnology, Santa Cruz, CA, USA), anti-insulin receptor (1∶2,000, MABS65, Millipore, Billerica, MA, USA), anti-BACE1 (1∶1,000, MAB5308, Millipore), anti-ADAM10 (1∶2,500, 422751, Calbiochem), anti-PS1 (1∶1,000, 529591, Calbiochem), an antibody that recognizes C-terminal epitope in APP (1∶1,000, C1/6.1, kindly provided by Dr. Paul Mathews, Nathan Kline Institute) to detect full-length APP/C-terminal fragments, anti-sAPPα (1∶500, 11088, Immuno-Biological Laboratories, Minneapolis, MN, USA), anti-phospho-eIF2α (Ser51) (1∶1,000, #3398, Cell Signaling Technology, Danvers, MA, USA), anti-eIF2α (1∶2,000, #9722, Cell Signaling Technology), anti-phospho-PERK (Ser713) (1∶500, #649401, BioLegend, San Diego, CA, USA), anti-cleaved caspase-3 (Asp175) (1∶1,000, #9661, Cell Signaling Technology), anti-GGA3 (1∶1,500, #4167, Cell Signaling Technology), anti-neprilysin 1∶2,000, ab951, Abcam, Cambridge, MA, USA), anti-IDE (1∶2,000, PC730, Millipore) or anti-β-actin (1∶15,000, AC-15, Sigma-Aldrich). They were then incubated with horseradish peroxidase-conjugated secondary IgG. Immunoblot signals were visualized by an ECL chemiluminescence substrate reagent kit (Pierce) and were quantified by densitometric scanning and image analysis using Quantity One software (Bio-Rad Laboratories, Hercules, CA, USA).

### ELISAs of Aβ40 and Aβ42

Sandwich Aβ ELISAs were performed as described previously [30], [31], [53]. Briefly, each hemibrain sample was extracted in 8X cold 5 M guanidine HCl plus 50 mM Tris HCl (pH 8.0) buffer, and centrifuged at 20,000 g for 1 h at 4°C to remove insoluble material. Final guanidine HCl concentrations were below 0.1 M. Protein concentrations were determined by a BCA kit (Pierce). To quantitate total levels of cerebral Aβ40 and Aβ42, supernatant fractions were analyzed by a well-established human Aβ40 and Aβ42 ELISA kits (KHB3481 and KHB3441, Invitrogen), respectively, according to the protocol of the manufacturer. Optical densities at 450 nm of each well were read on a VersaMax tunable microplate reader (Molecular Devices, Sunnyvale, CA, USA), and sample Aβ40 and Aβ42 concentrations were determined by comparison with the respective standard curves. Aβ40 and Aβ42 concentration values were normalized to total brain protein concentrations and expressed as the percentage of vehicle controls.

### Real-time qPCR

qPCR was performed in triplicate on frozen hemibrain samples as described previously [52], [54], [55]. TaqMan qPCR primers were utilized for mouse BACE1 mRNA (Mm00478671_m1, Applied Biosystems, Foster City, CA, USA) and the housekeeping gene glyceraldehyde-3-phosphate dehydrogenase (GAPDH, Mm99999915_g1, Applied Biosystems). Samples were assayed on a real-time qPCR cycler (7900HT, Applied Biosystems) in 96-well optical plates covered with optical adhesive film. Standard curves and cycle threshold were generated using standards obtained from total mouse brain RNA. The delta delta cycle threshold (ddCT) method was employed to determine relative gene level differences between STZ- and vehicle-treated 5XFAD mice with GAPDH qPCR products used as a control, and expression levels were presented as the percentage of vehicle controls. Negative controls consisted of the reaction mixture without input RNA.

### Statistical analysis

Significant differences between groups were determined by a one-way ANOVA and *post-hoc* Fisher's PLSD tests were performed when appropriate. Data were presented as mean ± SEM and the level of significance was set for *p* value less than 0.05.
